# Increase in HIV incidence in women exposed to rape

**DOI:** 10.1097/QAD.0000000000002779

**Published:** 2020-12-01

**Authors:** Naeemah Abrahams, Shibe Mhlongo, Kristin Dunkle, Esnat Chirwa, Carl Lombard, Soraya Seedat, Andre P. Kengne, Bronwyn Myers, Nasheeta Peer, Claudia Garcia-Moreno, Rachel Jewkes

**Affiliations:** a Gender and Health Research Unit, South African Medical Research Council; b Faculty of Health Sciences, School of Public Health and Family Medicine, University of Cape Town; c Biostatistics Unit, South African Medical Research Council, Cape Town; d Anxiety and Stress Disorder Unit, University of Stellenbosch, Stellenbosch; e Non-Communicable Diseases Research Unit, South African Medical Research Council; f Alcohol, Tobacco and Other Drug Research Unit, South African Medical Research Council; g Division of Addiction Psychiatry, Department of Psychiatry and Mental Health, University of Cape Town, Cape Town, South Africa; h Department of Sexual and Reproductive Health and Research, World Health Organization (WHO), Geneva, Switzerland; i Intramural Research Directorate, South African Medical Research Council, Cape Town; j Faculty of Health Sciences, School of Public Health, University of the Witwatersrand, Johannesburg, South Africa.

**Keywords:** HIV acquisition, HIV incidence, rape, sex-based violence, sexual violence, South Africa

## Abstract

**Objective:**

To determine the incidence of HIV acquisition in women postrape compared with a cohort of women who had not been raped.

**Design:**

A prospective cohort study.

**Methods:**

The Rape Impact Cohort Evaluation study based in Durban, South Africa, enrolled women aged 16–40 years from postrape care services, and a control group of women from Primary Healthcare services. Women who were HIV negative at baseline (441 in the rape-exposed group and 578 in the control group) were followed for 12–36 months with assessments every 3 months in the first year and every 6 months thereafter. Multivariable Cox regression models adjusted for baseline and time varying covariates were used to investigate the effect of rape exposure on HIV incidence over follow-up.

**Results:**

Eighty-six women acquired HIV during 1605.5 total person-years of follow-up, with an incident rate of 6.6 per 100 person-years [95% confidence interval (CI): 4.8–9.1] among the rape exposed group and 4.7 per 100 person-years (95% CI: 3.5–6.2) among control group. After controlling for confounders (age, previous trauma, social support, perceived stress, multiple partners and transactional sex with a casual partner), women exposed to rape had a 60% increased risk of acquiring HIV [adjusted hazard ratio: 1.59 (95% CI: 1.01–2.48)] compared with those not exposed. Survival analysis showed difference in HIV incident occurred after month 9.

**Conclusion:**

Rape is a long-term risk factor for HIV acquisition. Rape survivors need both immediate and long-term HIV prevention and care.

## Introduction

The intersecting and globally ubiquitous epidemics of rape and HIV are both partially driven by sex inequality [1,2]. This was demonstrated in a meta-analysis of four cohort studies included in a larger systematic review on the association between HIV and intimate partner violence (IPV) (*n* = 28 studies). Physical IPV [relative risk (RR): 1.22; 95% confidence interval (CI): 1.01–1.46] and any type of IPV (physical, sexual and psychological) (RR: 1.28; 95% CI: 1.00–1.64) were shown to be associated with greater HIV incidence [3]. The pathways by which IPV increases exposure to HIV infection in women include increased risk of direct transmission. Male partners who perpetrate IPV have more risky sexual behaviours, including multiple sexual partners and transactional sex, greater controlling behaviours, and more frequent substance use compared with nonviolent men. Pathways for indirect exposure to HIV infection include increases in subsequent risk behaviour and substance use among IPV survivors, often linked to adverse mental health outcome such as anxiety and depression [2,4–8].

Modelling studies in conflict settings [8,9] suggest a possible impact of rape on HIV incidence but the lack of data highlight the limitations of these epidemiological projections. To date, there are extremely limited observational data on the risk of HIV acquisition among women following exposure to rape. In particular, there are no data that elucidate on the possibility of greater HIV risk associated with the rape itself as well as the potential for sustained increases in HIV risk linked to the physical and psychosocial sequelae of experiencing rape. These co-occurring problems of rape and HIV infection are of particular concern for sub-Saharan Africa, which has the largest HIV burden globally [10], and high prevalence of IPV [11]. Unsurprisingly, there has been a call for more robust longitudinal studies to quantify the health burden of sexual violence in a range of settings and populations [1–3,6,12]. This article aims to address this gap by presenting findings from the first longitudinally study to assess the effects of rape on the incidence of HIV infection among women.

## Methods

### Study design

Women aged 16–40 years old were enrolled into a comparative cohort study, the Rape Impact Cohort Evaluation study. Women who attended postrape services after rape exposure (defined as nonconsensual penile-vaginal penetration) were included in the exposed group. Women in the control group were recruited from primary healthcare (PHC) clinics. Recruitment took place between October 2014 and April 2019 (42 months). Participants were followed for a minimum of 12 months and a maximum of 36 months, with final follow-up interviews conducted in March 2020. Assessment was done every 3 months in the first year and every 6 months thereafter to a maximum of eight follow-up interviews per participant. Ethical approval was granted by the South African Medical Research Council Ethics Committee (Protocol ID-EC019-10/2013). The full study design is reported elsewhere [13].

### Setting

The study was based in the greater Durban area in the KwaZulu-Natal Province of South Africa. Rape-exposed participants were recruited from five postrape service centres based at tertiary level public hospitals (four Thuthuzela Care Centers and a Crisis Clinic) [14]. Nonrape-exposed women were recruited from PHC clinics adjacent to the rape services; these were mainly Family Planning and Child Wellness services.

### Sample size calculation

Assuming a HIV incidence of 9 and 6% among the rape-exposed and nonexposed groups respectively [13], and to achieve 90% power to detect a significant difference in HIV incidence, 1008 participants were to be recruited in each cohort. In practice, however, recruiting rape-exposed participants took longer than anticipated resulting in logistical and budgetary constraints. Following consultation with the study statistician on the likely power required for the final analysis, recruitment was stopped in March 2019 and the last women enrolled were followed-up for 12 months. The final sample comprised of 852 rape-exposed and 947 nonrape-exposed women.

### Participant selection criteria

Women were eligible for inclusion in the rape-exposed cohort if the baseline interview took place within 20 calendar days of the index rape event. This was to ensure the temporal sequence of HIV testing and mental health assessments. Rape survivors who were severely mentally distressed or had cognitive disabilities were excluded. Women recruited at PHC clinics were screened for lifetime exposure to rape/forced sex and were excluded if childhood, intimate partner or nonpartner rape/forced sex were reported. Also excluded from both arms were women enrolled in other studies, more than 14 weeks pregnant or who were lactating, since HIV-positive lactating women are managed by different protocols.

### Retention of study participants

Participant follow-up was maintained through regularly updating of contact details and a retention counsellor assisted with barriers and logistics that is transport assistance. A trauma counsellor provided short-term counselling on request, including immediate counselling for those flagged during mental health assessments. Clinical staff provided pre and post-HIV test counselling and other health promotion (e.g. HIV-risk reduction). A home visitation program was initiated for those who missed follow-up visits. Participant reimbursements were incrementally increased by R20 ($US 1.2) per follow-up visit. Other initiatives to create a positive experience included a snack (sandwich) with tea and personal care services such as offering hand massages for women in the study waiting rooms.

### Variables and measurements

#### Biological investigations

We assessed HIV-1/2 serostatus using WHO recommended algorithms for HIV diagnosis in high prevalence settings [15]. An initial rapid test was performed with Alere Determine Rapid (Abbott Laboratories, Tokyo, Japan), with a confirmatory test using the FDA-approved Uni-Gold Recombigen HIV1/2 test (Trinity Biotech PLC, Wicklow, Ireland). A second confirmatory test was done on positive and equivocal results using the Abbot IMX ELISA (Abbot Laboratories, Africa Division) in combination with the Vironosktika HIV1/2 ELISA. Herpes Simplex (HSV2) was tested with Platelia HSV (1 + 2) IgG (Bio-Rad, Steenvoorde, France). *Trichomonas vaginalis* was tested with a self-collected vaginal swab using the OSOM Trichomonas Rapid Test (OSOM Sekisui Diagnostics (UK) Limited, Kent, UK) [16]. Participants who tested positive for *T. vaginalis* were treated onsite.

#### Sociobehavioural assessments

We collected questionnaire data via face-to-face interviews supported by a web-based data collection system. In 2017, we introduced self-completion of aspects of follow-up interviews. Sociodemographic data included age, education (</>12 years), employment status (full-time/part-time/self-employment), reliance on a government Child Support Grant for children in low-income households, settlement type (formal-urban/informal-urban/rural) and food insecurity as a proxy measure for poverty. Food insecurity was assessed by how often people in the home went without food, (never/seldom or sometimes/often). Community social support was measured by how easy or difficult it was to borrow money [R200 (US$ 12)] for an emergency (easy/very easy or quite difficult/very difficult) [17].

#### Sexual reproductive health and sexual risk behaviours

Sexual behavioural measures included ever having had consensual sex, age at first sex (</>16 years), condom use in past year (always/often or sometimes/never) and pregnancy history. Self-reported history of a sexually transmitted infection (STI) was derived from three questions: ever having had a vaginal discharge/vaginal ulcers or told by a health worker they had an STI. If the rape-exposed women received postexposure prophylaxis (PEP) for the prevention of HIV, self-reported adherence was assessed at the 3-month or next follow-up visit.

Intimate relationships [currently not in a relationship/married or cohabiting/dating (not cohabiting), number and type of sexual partners (main/casual), history of transactional sex with partners (ever/past year)] were assessed using measures derived from South African studies [17,18].

#### Violence and mental health measures

The widely used South African version of the WHO Multi-Country Study measured ever and past 12-month IPV (emotional/economic/physical/sexual) and nonpartner sexual violence [19]. We assessed control in intimate relationships using the South African adaptation of the *Relationship Control Scale*[20]. Previous trauma was measured using a modified *Life Events Checklist*[21] and childhood trauma (before the age of 18 years) was measured using a modified version of the *Childhood Trauma Questionnaire–Short Form*[22]. Sexual violence reported in the latter two scales was important to ensure participants had no previous exposure to rape as per study exclusion criteria. The *Davidson Trauma scale* assessed Post Traumatic Stress Symptoms [23]. The *Center for Epidemiologic Studies Depression Scale* examined current depression symptomatology [24]. The *Multidimensional Scale of Perceived Social Support* measured social support [25] and the *Perceived Stress Scale* measured perceived stress [26] with a high score indicating better support or greater stress. A brief version of the *Alcohol Use Disorders Identification Test* (AUDIT), the AUDIT-C, was used to measure alcohol use with high score indicating risky alcohol use [27]. These scales have all been used widely in South Africa.

### Statistical analysis

Characteristics of all HIV-negative participants at baseline including sociodemographic, intimate partner and nonpartner violence experience, risky sexual behaviour and psychosocial factors were summarized using means and SD or frequencies with percentages. We used the *t* test for continuous variables and the Pearson's Chi-square test for categorical variables to assess associations between baseline characteristics and loss-to-follow-up within each exposure group. To assess whether baseline characteristics differentially impacted on loss-to-follow-up between the two groups, we performed a series of logistic regressions with loss-to-follow-up as the outcome and tested for significant statistical interaction between each baseline characteristic and the exposure group. We next compared the baseline characteristics of the women who acquired HIV and those who did not during follow-up using the *t* test for continuous variables and the Pearson's chi square for the categorical variables.

Including all women with follow-up data, we calculated incidence rates of HIV for the two groups and summarized the person-years. Time to event analysis was conducted to investigate the effect of rape exposure on HIV acquisition. For participants who sero-converted, time-to-event was measured from time they enrolled into the study to the time of first positive HIV result. Time-to-event was censored at the last visit for those who remained HIV negative or were loss-to-follow-up.

The data were analysed using a Cox proportional hazard model after assessing for the proportional hazard assumption. Predetermined baseline covariates included in the model were participant age and life-time experience of traumatic events. Factors found to be associated with differential loss-to-follow-up by exposure group (social support and perceived stress) were also included. Trajectories of time varying covariates describing key sexual risk behaviour (multiple partners and transactional sex with a casual partner) in the follow-up period were assessed (shown in Supplemental Digital Contents 3 and 4). A time-varying composite variable with three levels of sexual risk behaviour (low/moderate/high) based on combining multiple partners and transactional sex with casual partner within each follow-up period was derived to reflect changes in sexual risk behaviour over time. The final model was adjusted for this time-varying composite variable. Similarly constructed Weibull models were used as confirmatory models (see Supplemental Digital Content 3).

We conducted several additional sensitivity analyses presented in the Supplementary files to check the robustness of results under different assumptions for missing data and possible HIV infection before and during the rape. We looked at the impact of excluding participants based on when they dropped out of the study (at 3, 6 or 9 months) for both the main analysis model (Cox regression) and the confirmatory model (Weibull), adjusting for the same covariates. Sensitivity analysis included taking into account the possibility of HIV infections before and during the rape, and we constructed a model shifting the starting point to month 3. We also fitted models excluding women in the control group who reported previous rape exposure (but did not disclose during their screening interview at baseline). Data were analysed using STATA 16 (Statacorp LLC, College Station, Texas, UK).

## Results

Of the 1799 participants enrolled, 1019 were HIV negative at baseline and included in this analysis: 441 in the rape-exposed group and 578 in the control group (Fig. S1: see flow diagram and text presented in Supplementary content 1). There was a significant difference in retention (*P* < 0.001) between the two groups at 6 months with lower retention (63.5%; *n* = 280/441) among rape-exposed participants vs. among controls [80.1% (*n* = 463/578) (Table 1)]. None of the sociodemographic, behavioural or psychosocial factors assessed at baseline were significantly associated with loss-to-follow-up among the rape-exposed group. Among the control cohort, loss-to-follow-up vs. retained participants, reported fewer IPV experiences at baseline, more social support and less stress. Moreover, baseline levels of the latter that is social support and perceived stress impacted more significantly on loss-to-follow-up in control vs. rape-exposed participants (*P* = 0.009 and 0.042, respectively).

**Table 1:** Baseline sociodemographic and behavioural characteristics among participants retained and lost to follow-up at 6 months by exposure group (*N* = 1019).

	Rape-exposed, *n* = 441			Unexposed (control group), *n* = 578			
	Retained, *n* = 280 (63.5%)	Lost to follow-up, *n* = 161 (36.5%)		Retained, *n* = 463 (80.1%)	Lost to follow-up, *n* = 115 (19.9%)		Differential impact on loss to follow-up between groups
	*n* (%) or mean (SD)		*P* value^a^	*n* (%) or mean (SD)		*P* value^a^	*P* value^b^
Age (years)	23.1 (4.7)	23.2 (4.7)	0.786	24.0 (4.6)	23.2 (4.2)	0.101	0.157
Education grade 12 and more	191 (68.2%)	113 (70.2%)	0.667	326 (70.4%)	87 (75.7%)	0.265	0.589
Employed	62 (22.1%)	47 (29.2%)	0.098	63 (13.6%)	15 (13.0%)	0.874	0.271
Currently in an intimate relationship^c^	221 (78.9%)	123 (76.4%)	0.537	389 (84.2%)	102 (88.7%)	0.226	0.181
IPV	173 (61.8%)	85 (52.8%)	0.065	258 (55.7%)	51 (44.4%)	0.029	0.760
NPSV^c^	4.5 (1.0)	4.5 (1.0)	0.605	4.1 (0.3)	4.1 (0.3)	0.671	0.799
Relationship control scale	20.9 (6.4)	21.3 (6.8)	0.487	21.4 (5.3)	22.1 (5.0)	0.178	0.487
HSV2 positive	156 (55.7%)	83 (51.6%)	0.398	254 (54.9%)	52 (45.2%)	0.064	0.447
Transactional sex	24 (8.6%)	15 (9.3%)	0.791	41 (8.9%)	6 (5.2%)	0.201	0.245
Multiple partners	48 (17.1%)	28 (17.4%)	0.947	50 (10.8%)	12 (10.4%)	0.910	0.896
STI ever^c^	122 (43.6%)	56 (34.8%)	0.13	181 (39.1%)	48 (41.7%)	0.615	0.106
Social support^d^	35.2 (5.1)	34.8 (5.7)	0.464	34.9 (4.6)	36.2 (4.4)	0.005	0.009
Perceived stress^e^	22.9 (5.7)	23.3 (6.4)	0.539	21.5 (5.0)	20.4 (5.0)	0.035	0.042
Previous experiences of trauma	1.9 (1.7)	1.8 (1.7)	0.311	1.1 (1.4)	0.97 (1.2)	0.547	0.891
Childhood trauma	16.6 (3.6)	16.7 (3.9)	0.793	16.0 (2.8)	15.7 (2.9)	0.368	0.371
Audit C score^c^	2.1 (2.6)	1.8 (2.4)	0.232	1.6 (2.3)	1.4 (2.3)	0.417	0.870
PTSS-DTS^c^	35.3 (15.9)	33.7 (15.5)	0.317	6.4 (9.8)	5.4 (9.1)	0.322	0.695
Depression-CESD^c^	33.2 (12.6)	33.2 (12.5)	0.964	13.3 (9.1)	11.9 (8.6)	0.139	0.221

CESD, Center for Epidemiological Studies Depression; DTS, Davidson trauma scale; HSV2, herpes simplex virus type 2; IPV, intimate partner violence-ever experienced emotional, economical, sexual or physical violence; NPSV, non-partner sexual violence; PTSS, post-traumatic stress syndrome; STI, sexually transmitted infection.

a Pearson's chi-square and *t* test were applied to compare the baseline characteristics between the retained and the loss to follow-up within each exposure group.

b The logistic regression was applied to obtain the interaction term effect of the baseline characteristic and the exposure group on the loss to follow-up outcome.

c *N* = 1018, with retained total *n* = 742 and unexposed retained *n* = 462.

d *N* = 1016, with retained total *n* = 740 and unexposed retained *n* = 460.

e *N* = 1017, with retained total *n* = 741 and unexposed retained *n* = 461.

Eighty-six women acquired HIV during 1605.5 total person-years of follow-up: 37 in the exposed group and 49 in the unexposed group (Table 2). Incident rates of 6.6 per 100 person-years (95% CI: 4.8–9.1) among the rape-exposed group and 4.7 per 100 person-years (95% CI: 3.5–6.2) among the control group were found for those retained after baseline. We also summarized the person-years pre- and post-12 months (see Table S1 in Supplementary content 2). Overall, few significant differences were found between women who acquired HIV and those who did not (Table 3). The mean age at study enrolment was similar [incident HIV: 23.1 years; no incident HIV: 23.6 years (*P* = 0.383)]. However, women who acquired HIV were less educated (59.3 vs. 71.4%: *P* = 0.019), and reported more physical IPV in the previous year at baseline (20.9 vs. 12.7%: *P* = 0.031), but were less likely to have experienced sexual abuse as a child (4.7 vs. 13.0%: *P* = 0.026).

**Table 2:** HIV incidence among all participants who were HIV negative at baseline by exposure group for 3, 6 and 9 months follow-up (*N* = 845).

	Exposure group	Number of sero-converters	Person-years	Incidence rate per 100 person-years (95% CI)
All participants retained after baseline (*n* = 845)	Unexposed	49	1046.25	4.7 (3.5–6.2)
	Rape-exposed	37	559.25	6.6 (4.8–9.1)
			1605.50^∗^	
Retained from Month 3 (*n* = 783)	Unexposed	49	1039.50	4.7 (3.6–6.2)
	Rape-exposed	37	550.75	6.7 (4.9–9.3)
			1590.25^∗^	
Retained from Month 6 (*n *−* *742)	Unexposed	49	1029.50	4.8 (3.6–6.3)
	Rape-exposed	37	540.25	6.8 (5.0–9.5)
			1569.75^∗^	
Retained from Month 9 (*n *−* *715)	Unexposed	49	1021.25	4.8 (3.6–6.3)
	Rape-exposed	37	529.00	7.0 (5.1–9.7)
			1550.25^∗^	

CI, confidence interval.

∗ Total person-years.

**Table 3:** Baseline sociodemographic, sexual behaviour, relationship and previous trauma characteristics associated with HIV incidence (*N* = 1019).

	Incident HIV: *n* = 86 (8.4%)		No incident HIV: *n* = 933 (91.6%)		
	*n* (%) or mean (SD)	95% CI	*n* (%) or mean (SD)	95% CI	*P* value^a^
Demographics					
Age (years)	23.1 (4.6)	22.1–24.1	23.6 (4.6)	23.3–23.9	0.383
Education grade 12 and more	51/86 (59.3%)	48.6–69.2	666/933 (71.4%)	68.4–74.2	0.019
Employed	12/86 (14.0%)	8.1–23.0	175/933 (18.8%)	16.4–21.4	0.271
Income from child support grant (among women with children who are alive)	29/60 (48.3%)	36.0–60.8	251/596 (42.1%)	38.2–46.1	0.353
Settlement area					
Formal/Urban	58/85 (68.2%)	57.6–77.2	683/922 (74.1%)	71.1–76.8	0.218
Informal/Urban	14/85 (16.5%)	10.0––25.9	152/922 (16.5%)	14.2–19.0	
Rural	13/85 (15.3%)	9.1–24.6	87/922 (9.4%)	7.7–11.5	
Food security					
Sometimes and often the home goes without food	12/86 (14.0%)	8.1–30.0	157/932 (16.9%)	14.6–19.4	0.490
Finding money for an emergency					
Very and quite difficult to find money in an emergency	76/86 (88.4%)	79.9–93.6	774/933 (83.0%)	80.4–85.2	0.196
Sexual behaviours					
Ever had sex at baseline	82/85 (96.5%)	89.6–98.9	881/933 (94.4%)	92.8–95.7	0.425
Age at first sex (≤15 years)	11/86 (12.8%)	7.2–21.7	77/933 (8.3%)	6.7–10.2	0.357
Ever pregnant at baseline	63/85 (74.1%)	63.8–82.3	640/933 (68.6%)	65.5–71.5	0.292
Ever transactional sex with main partner	7/86 (8.1%)	3.9–16.1	75/933 (8.0%)	6.5–10.0	0.974
Ever transactional sex with casual partner	11/86 (12.8%)	7.2–21.7	75/933 (8.0%)	6.5–10.0	0.129
Sexually transmitted infection ever	35/85 (41.2%)	31.2–51.9	372/933 (39.9%)	36.8–43.1	0.814
Has had 5+ partners ever	21/85 (24.7%)	16.7–35.0	173/931 (18.6%)	16.2–21.2	0.169
Has had 2+ partners in the last year	18/86 (20.9%)	13.6–30.8	120/933 (12.9%)	10.9–15.2	0.036
Rarely use condoms used in past year	35/85 (41.2%)	31.2–51.9	465/932 (49.9%)	46.7–53.1	0.124
Relationship at baseline					
Not in a current relationship	19/85 (22.4%)	14.7–32.4	164/933 (17.6%)	15.3–20.2	0.294
Cohabiting/married	3/85 (3.5%)	1.1–10.4	66/933 (7.1%)	5.6–8.9	
Dating not cohabiting	63/85 (74.1%)	63.8–82.3	703/933 (75.4%)	72.5–78.0	
IPV					
Emotional IPV ever	40/86 (46.5%)	36.3–57.1	378/933 (40.5%)	37.4–43.7	0.279
Emotional IPV in last 12 months	15/86 (17.4%)	4.1–2.7	121/933 (13.0%)	11.0–15.3	0.243
Economic IPV ever	16/86 (18.6%)	11.7–28.2	145/933 (15.5%)	13.3–18.0	0.456
Economic IPV past 12 months	5/86 (5.8%)	2.4–13.2	64/933 (6.9%)	5.4–8.7	0.712
Physical IPV ever	43/86 (50.0%)	39.6–60.4	387/933 (41.5%)	38.4–44.7	0.126
Physical IPV past 12 months	18/86 (20.9%)	13.6–30.8	118/933 (12.7%)	10.7–14.9	0.031
Sexual IPV ever	12/86 (14.0%)	8.1–23.0	118/933 (12.7%)	10.7–14.9	0.728
Sexual IPV past 12 months	5/86 (5.8%)	2.4–13.2	51/933 (5.5%)	4.2–7.1	0.892
NPSV	4.2 (0.6)	4.1–4.4	4.3 (0.7)	4.2–4.3	0.953
Ever IPV and NPSV	59/86 (68.6%)	58.1–77.5	568/933 (60.9%)	57.7–64.0	0.159
Relationship control-scale score	20.9 (5.3)	19.8–22.1	21.4 (5.9)	21.0–21.7	0.506
Previous trauma					
Previous experiences of trauma score	1.3 (1.4)	1.0–1.5	1.4 (1.6)	1.3–1.5	0.404
Experienced two or more previous trauma	31/86 (36.1%)	26.6–46.7	337/933 (36.1%)	33.1–39.3	0.989
Childhood trauma					
Childhood trauma score	15.6 (2.4)	15.1–16.1	16.3 (3.3)	16.1–16.5	0.063
Experienced 2 or more childhood traumas	27/86 (31.4%)	22.5–41.9	356/933 (38.2%)	35.1–41.3	0.215
Experienced sexual abuse as a child	4/85 (4.7%)	1.8–11.9	121/933 (13.0%)	11.00–15.3	0.026
Experienced physical abuse as a child	29/86 (33.7%)	24.5–44.3	414/933 (44.4%)	41.2–47.6	0.057
Experienced emotional abuse/neglect as a child	30/86 (34.9%)	25.6–45.5	336/933 (36.0%)	33.0–39.2	0.835
Alcohol use					
Audit C score	1.8 (2.5)	1.2–2.3	1.7 (2.4)	1.6–1.9	0.905

CI, confidence interval; IPV, intimate partner violence; NPSV, non-partner sexual violence.

a Pearson's chi-square and t test were applied to compare the baseline characteristics for the HIV incidence outcome.

**Fig. 1:**
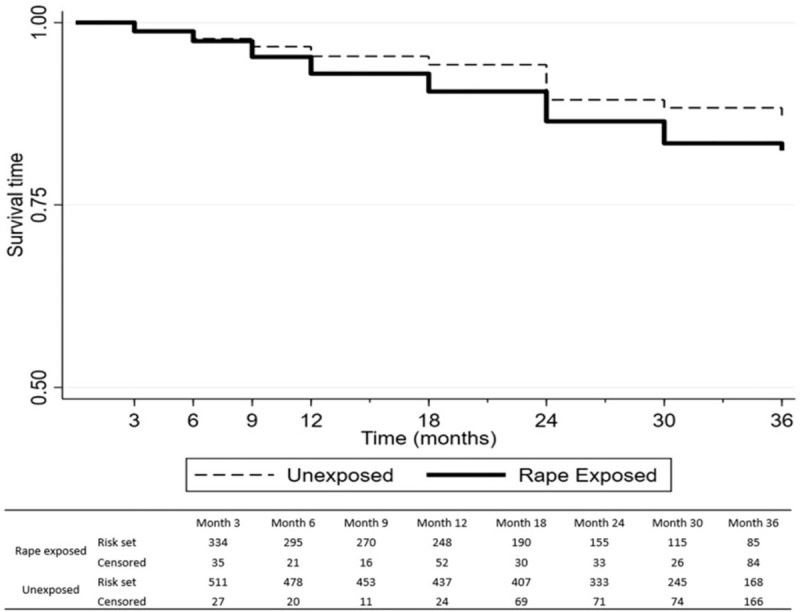
Kaplan–Meier survival plot depicting HIV sero-conversion by month of follow-up: comparing recently rape-exposed women with nonexposed control women.

The Kaplan–Meier survival plot of HIV acquisition over time across the two study arms (Fig. 1) suggested that there was no significant difference in rape incidence until after month 9, after which women exposed to rape were more likely to seroconvert than nonexposed women. Multivariable Cox regression models adjusted for variables associated with differential loss to follow-up (social support and perceived stress), for baseline covariates (age and previous trauma exposure) and time varying covariates known to be associated with HIV risk among South Africa women (multiple sexual partners and transaction sex) showed women recently exposed to rape at study enrolment were nearly 60% more likely to acquire HIV compared with the women not exposed to rape (Table 4), with a hazard ratio of 1.59 in the fully adjusted model (95% CI: 1.01–2.48: *P* = 0.043). A further series of comprehensive sensitivity analyses showed the finding of sustained increase in HIV risk among women exposed to rape to be consistently robust to a range of analytic approaches shown in the sensitivity analysis presented in the supplementary file (Tables S2–S7 with text on sensitivity analysis in Supplementary content 3 and Figs. S2–S4 shown in Supplementary content 4,).

**Table 4:** Cox regression models of relative incidence of HIV amongst all those retained after baseline adjusted for variables associated with drop-out, baseline covariates and time varying covariates (*N* = 845).

	Hazard ratio (95% CI)	*P* value
Unadjusted model (*N* = 845)		
Unexposed	1.00	–
Rape-exposed	1.42 (0.93–2.18)	0.108
Adjusted for baseline covariates (*N* = 845)		
Adjusted for baseline age		
Unexposed	1.00	–
Rape-exposed	1.39 (0.91–2.14)	0.131
Adjusted for baseline age and previous trauma experience reported at baseline		
Unexposed	1.00	–
Rape-exposed	1.48 (0.95–2.31)	0.085
Adjusted for baseline covariates (age and previous trauma experiences) and time-varying covariates (*N* = 845)		
*Adjusting for baseline covariates and time varying covariate: multiple partners in follow-up period*^a^		
Unexposed	1.00	–
Rape-exposed	1.57 (1.00–2.44)	0.04
*Adjusting for baseline covariates and time varying covariate: transactional sex with casual partner in follow-up period*^b^		
Unexposed	1.00	–
Rape-exposed	1.60 (1.03–2.50)	0.037
*Adjusting for baseline and time varying covariates: combined measure of multiple partners and transactional sex with casual partner (3 levels)*^c^		
Unexposed	1.00	–
Rape-exposed	1.58 (1.01–2.47)	0.043
Adjusted for baseline covariates (age and previous trauma experiences) and variables associated with drop out (social support and perceived stress) and time varying covariates (combined multiple partners in past year and transactional sex with casual partner) (*N* = 842)		
Unexposed	1.00	–
Rape-exposed	1.59 (1.01–2.48)	0.043

CI, confidence interval.

a Reported having two or more sexual partners in the period between interviews.

b Reported having transactional sex with a casual partner in the period between interviews.

c A three levels composite sexual risk behaviour variable (low, moderate and high) based on combining multiple partners and transactional sex with casual partner in the follow-up period.

## Discussion

Our data show that rape is associated with HIV acquisition among women in South Africa. Importantly, we show that exposure to rape increases risk of HIV acquisition over an extended period postrape, rather than being primarily driven by the rape event itself. To our knowledge this is the first longitudinal study of recent rape with a control group to demonstrate such an association. The possibility of direct transmission during the rape event should be considered given that there may have been genital trauma which would have increased the likelihood of HIV transmission [28]. However, we have no information on genital injury or the HIV status of the perpetrators. Four (4/37) rape-exposed women were diagnosed with HIV infection at their 3-month follow-up visit, but it is uncertain whether these women were exposed to HIV before, during, or after the rape. All four women reported receiving PEP postrape and taking the full 28 days of treatment. More importantly, sensitivity analysis using the 3-month postenrolment as the starting point confirms that direct transmission after rape was very unlikely (Table S7 in Supplementary content 3).

The excess seroconversions among rape-exposed women outside the 3-month window in which they could be reasonably attributed to the rape itself must be due to indirect pathways stemming from increases in other risk factors following the experience of rape. This is analogous to the multiple indirect pathways from IPV to HIV acquisition noted in numerous studies in a wide range of settings [2,3]. Similar indirect pathways are likely post rape, including increasingly poor mental health leading to increases in risky behaviour including multiple, concurrent, transactional sexual partners, low condom usage and increased substance use [2]. A study among female sex workers found alcohol abuse to be associated with sexual violence in the 12-month follow-up period [29]. Understanding these potential pathways will require further exploration of the longitudinal dataset such as the role of mental health outcomes.

The long-term impact of rape on HIV acquisition affirms the potential importance of pre exposure prophylaxis (PrEP) as a biobehavioural intervention for rape survivors [30]. A recent scoping review on the potential benefits of PrEP for IPV survivors shows that this is an emerging area of research [31] and the full benefit of this women-centred approach has not received attention. To our knowledge PrEP is not offered as part of post rape care in South Africa and our study finding increases the urgency to better understand the role of PrEP to prevent HIV among both IPV and rape survivors.

Biological changes are another possible link between rape and prolonged increase in HIV risk. Despite extensive knowledge on the immunity of the female reproductive tract and how inflammatory responses increase the likelihood of HIV acquisition, very little is known about how experiences of violence and rape impact immunity. A small body of emerging research on the immune mechanisms between chronic sexual violence, mental health and HIV susceptibility suggests possible changes in the immunological environment of the genital mucosa [32]. Earlier research on a small cohort of HIV-positive women measured both CD4^+^ and CD8^+^ and showed that reduction in CD8^+^ decline was 70.5% higher in sexually abused women than those who did not report sexual abuse [33]; although this finding was not statistically significant due to small sample size, it nonetheless points to a possible impact of rape on women's immunity. This warrants future investigation into the inflammatory responses associated with rape and trauma.

The study has strengths and limitations. There were some differences between the groups at baseline such as age and employment but not education and food security (Table S2 in the Supplementary content 3). Both groups however were within the same age risk profile (mean age: 23.2 vs. 23.8) for HIV infection and we controlled for age within the analysis. Moreover, our findings cannot be generalized to women outside the 16–40-year age group, or outside South Africa, nor can it be generalized to men, transgender or nonbinary people. We used a robust measure (WHO protocol) to test for HIV at each follow-up visit, mirroring diagnostic approaches used in South African. We considered a control arm of women who utilised PHC services would have more similarities with women who sought postrape care, rather than a control group from the general population. Any population seeking healthcare (either postrape care or clinic care) may pay more attention to personal health than the general public and this might impact their risk for HIV acquisition. In a context where rape is seriously under-reported [34], it is likely that women who present at postrape care services are different from those who do not, which may lead to their differential risk for HIV acquisition that is seeking postrape care to access PEP.

Retention in the rape-exposed group was significantly lower than the unexposed group, however, no measured factors associated with HIV incidence were associated with loss-to-follow-up in this group, which mitigates the difference. Follow-up data on HIV acquisition were interval censored, but we employed standard methods for dealing with uncertain time of seroconversion, and this uncertainty was nondifferential between the study arms.

While there may have been unmeasured factors which impacted on HIV acquisition among participants, we have measured and adjusted for the most common behavioural HIV risk factors among women in the study setting. A few set of studies have found a history of child sexual abuse associated with HIV risk behaviour [35], however, previous exposure to rape, including child rape were exclusion criteria for the nonexposed group, which prevented adjustment for child sexual abuse. We also considered including the *T. vaginalis* test as an indicator of STI; however, missing data from menstruating participants prevented this analysis.

### Conclusion

The current study shows rape is associated with a prolonged increase in the risk of HIV infection. Clinical protocols for postrape care must be updated, as the current focus on providing PEP is insufficient to fully prevent HIV acquisition in the longer term. Rape survivors need long-term follow-up care focusing on their overall well being, psychosocial health and sexual health. Providing this care is complex, as rape survivors are vulnerable to dropping out of care that may remind them of the trauma, a phenomenon seen especially in poor rates of PEP completion [36]. This challenge must be addressed through comprehensive, extended, compassionate care that includes psychosocial support, or our postrape HIV prevention efforts are likely to fail.

## Acknowledgements

Author contribution: Conceptualization was led by N.A. and R.J. with input from all authors. Project implementation and management was done by N.A. Data curation was done by S.M., C.L. and E.C. Formal analysis was done by S.M., E.C., C.L., N.A., R.J. and K.D. with initial interpretation done by same team. The original draft was written by N.A. and all authors contributed to writing, reviewing and editing the article.

We thank the participants for their time and sharing information. We thank the National Prosecuting Authority and the KwaZulu-Natal Department of Health for their support. We thank Prince Mshiyeni, R K Khan, Mahatma Gandhi and Tongaat Hospitals Thuthuzela Care Centres and the Addington Hospital Crisis Centre management and staff for their assistance. We also thank the following research staff: Sinqobile Mngadi, Tholsie Gounden, Thobeka Majola, Sanelisiwe Ntombela, Mpumelelo Mabhida, Zandile Ngcobo, Nokwazi Ntuli, Melda Magolela, Veronica Chamane, Khanyisile Ngcingwana, Alesha Sewnath, Janice Julius, Thamsanqua Tokota, Bongumusa, Mthembu, Nikiwe Ntanzi, Rebecca Ntanzi, Phindile Kheswa, Prilene Stroud, Thobile Majola, Pearl Mdlalose, Ayanda Tembe, Nokukhanya Nzimande, Hlengiwe Lukhashe, Winnie Hlophe, Matimba Baloyi and Praysgod Ndlovu.

The current research and the publication thereof is the result of funding provided by the South African Medical Research Council in terms of the SAMRC's Flagships Awards Project SAMRC-RFA-IFSP-01-2013/RAPE COHORT.

Funding: South African Medical Research Council: Flagships Awards Project SAMRC-RFAIFSP-01-2013.

### Conflicts of interest

C.G.M. is a staff member of WHO. The authors alone are responsible for the views expressed in this publication and they do not necessarily represent the decisions or policies of WHO. We declare there are no conflicts of interest.

## Supplementary Material

Supplemental Digital Content

## Supplementary Material

Supplemental Digital Content

## Supplementary Material

Supplemental Digital Content

## Supplementary Material

Supplemental Digital Content
