# Supplementary material for: Increase in HIV incidence in women exposed to rape
Source: AIDS. 2020 Dec 1;35(4):633–42. doi: 10.1097/QAD.0000000000002779 (PMC7924974; doi:10.1097/QAD.0000000000002779)
Supplement: Supplemental Digital Content [file aids-35-633-s001.docx]

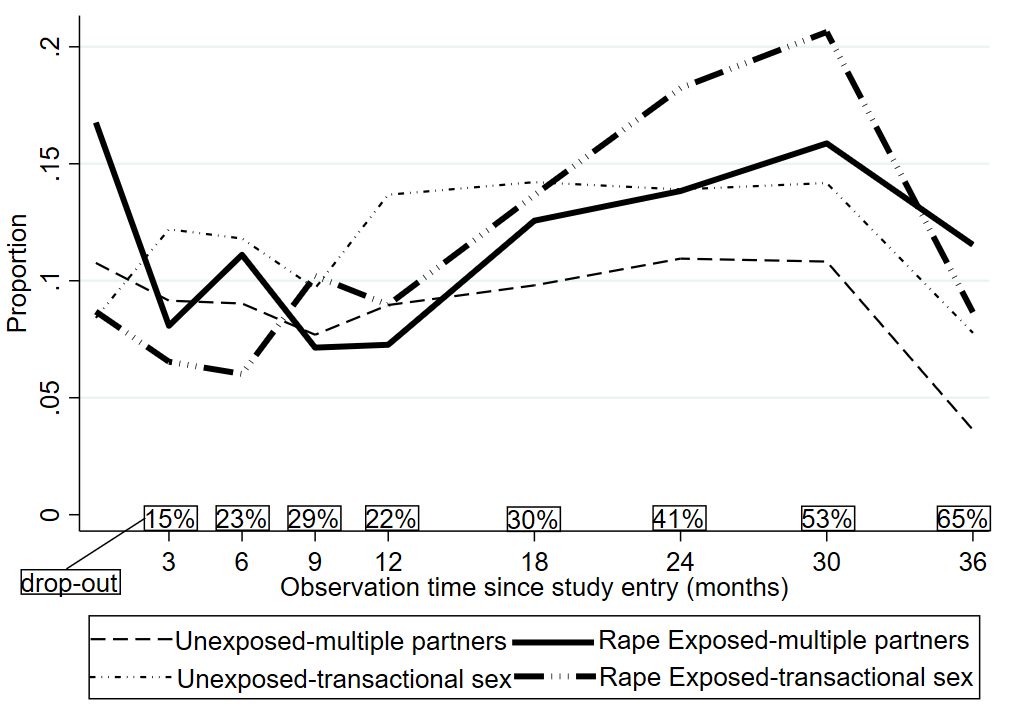


Figure S2: Graph depicting transition of multiple partners and transactional sex over time by exposure group


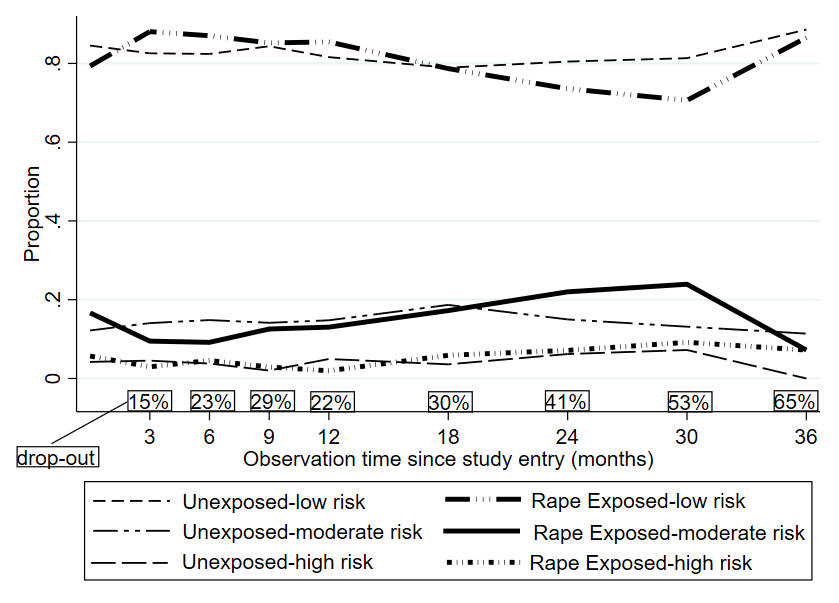


Figure S3: Graph depicting transition of composite risk variable over time by exposure group


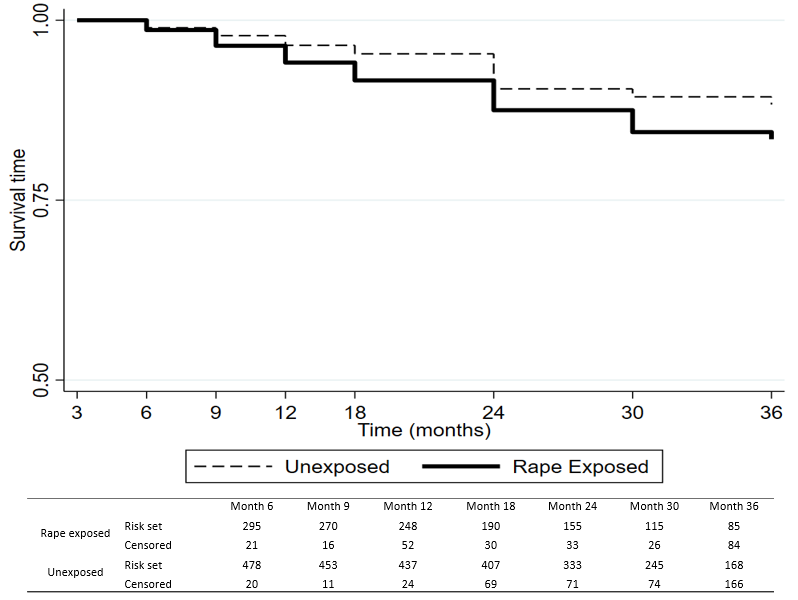


Figure S4: Kaplan-Meier survival plot depicting HIV sero-conversion by month of follow up with starting time shifted to month 3 by exposure group
