# Supplementary material for: Increase in HIV incidence in women exposed to rape
Source: AIDS. 2020 Dec 1;35(4):633–42. doi: 10.1097/QAD.0000000000002779 (PMC7924974; doi:10.1097/QAD.0000000000002779)
Supplement: Supplemental Digital Content [file aids-35-633-s002.docx]

Figure S1: Flow diagram showing enrollment and retention of the Rape Impact Cohort Evaluation (RICE) study participants


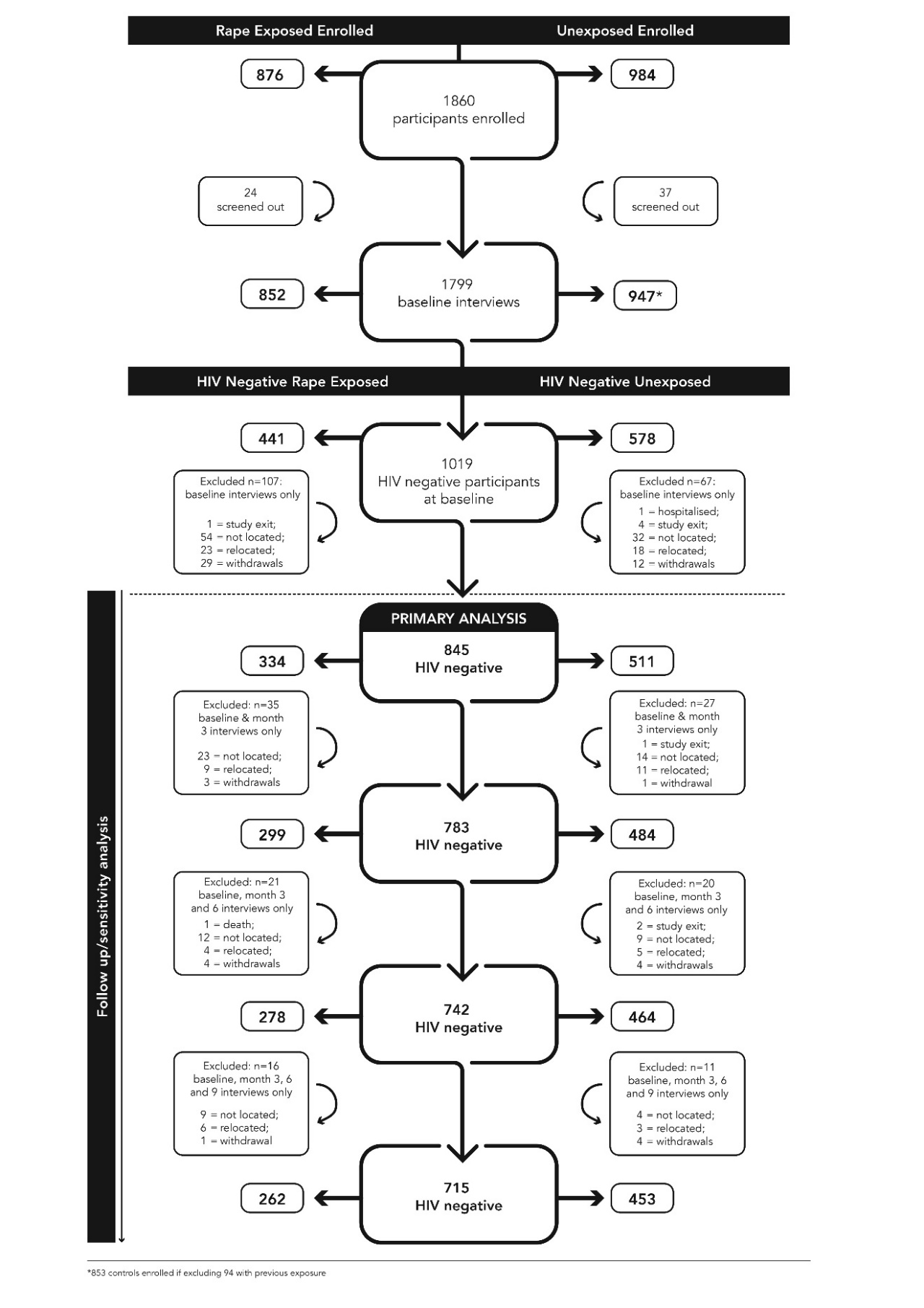


**Explanation of study flow**

The enrollment, arm allocation, loss to follow-up and reasons for non-retention are presented in Figure S1. A total of 1860 participants were enrolled but 61 were screened-out after registration on the web-based data collection system: 24 rape exposed women were found to have experienced the rape event outside of the study time-period, and 37 unexposed participants reported previous exposure to rape. Later in the study, despite screening procedures, 94 control arm participants disclosed experiences of rape before enrollment. These women were included in the analysis as per published protocol. Among the remaining 1799 participants, 1019 were HIV negative at baseline. As 107 HIV negative exposed women and 67 control participants only completed a baseline interview, these participants were excluded from longitudinal analyses. The most common reason for loss-to-follow-up was inability to trace them (54 in the exposed group and 32 in the unexposed group). Twenty-nine of the exposed participants withdrew compared to12 participants from the control arm; 23 of the exposed group and 18 of the unexposed group relocated to distant rural areas or other provinces, and four participants in control arm and one in the exposed arm were found to have co-enrolled in another study (study exit). We had one serious adverse event (death due to suicide) reported at 9-months among the rape exposed women and one adverse event among the unexposed group (hospitalisation). The primary analysis assessing HIV incidence was done on 845 participants.
