# Supplementary material for: Increase in HIV incidence in women exposed to rape
Source: AIDS. 2020 Dec 1;35(4):633–42. doi: 10.1097/QAD.0000000000002779 (PMC7924974; doi:10.1097/QAD.0000000000002779)
Supplement: Supplemental Digital Content [file aids-35-633-s003.docx]

**Sensitivity analysis**

A series of sensitivity analysis were done to assess the robustness of the findings to various decisions on how to handle differential loss-to-follow-up (after 3,6, 9 months), whether excluding the HIV negative women among the unexposed group who were ultimately identified as having been previously raped (after initial screening) and shifting the starting point to month 3 thus excluding participants who sero-converted at the 3 month follow-up visit. The following additional analysis were done

- We started by exploring socio demographic differences between exposure groups to identify baseline covariates associated with exposure (Table S2).
- We next explored differential loss-to-follow-up at 3,6 and 9 months across the two cohorts. We show similar findings across the three follow-up periods. We present the results of the analysis among those retained after six months or longer in the main paper (Table 2) and the three (Table S3) and nine months (Table S4) in the supplementary file. We subsequently adjusted for the two identified variables (social support and perceived stress) in all the HIV incidence models.
- We also explored key risk behaviour characteristics i.e. multiple partners and transactional sex with a casual partner in the follow-up period. The trajectories over time were explored using transition tables (Fig. S2). The constructed sexual risk behavior composite variable was also explored (Fig. S3). The composite variable was adjusted in all final models.
- We calculated HIV incidence for all participants and for the different retention groups (shown in Table 2) and for follow-up pre-12 months and post-12 months (Table S1). This analysis shows minimal differences.
- We constructed a confirmatory survival analysis model using the Weibull model (Table S5) applying the same adjustments as for the Cox regression model (Table 4). The two survival models how similar results for HIV acquisition following rape.
- We then did an analysis to determine if the outcome of the survival analyses would differ if we excluded the HIV negative women from the unexposed group who were found to have been exposed to rape previously (Table S6). We constructed both a Cox regression and a Weibull model and based on the results included all HIV negative women from the unexposed (control) group in our main analysis.
- To exclude HIV infections before the index rape (rape exposed arm) or baseline interview (control arm) we shifted the starting time to month 3 to exclude participants that dropped out after 3 months and seroconverts at 3 months (Table S7). We found similar results to the analysis done from baseline. We also present this analysis in a Kaplan Meier graph (Fig. S4).

All the of the above additional analysis confirmed the robustness of our findings.

**Table S2: Socio-demographic characteristics of all HIV negative participants at baseline (N=1019) by exposure group**

|  | **HIV negative participants**  **n= 1019** | | | **Rape-exposed**  **n =441 (43.3%)** | | | **Unexposed (Control Group)**  **n =578 (56.7%)** | | |  |
| --- | --- | --- | --- | --- | --- | --- | --- | --- | --- | --- |
|  | n | %/ mean (sd) | 95% CI | n | %/ mean (sd) | 95% CI | n | %/ mean (sd) | 95% CI | *P* value ^a^ |
| Age (mean (sd)) | 1019 | 23.5 (4.6) | 23.2-23.8 | 441 | 23.2 (4.7) | 22.7-23.6 | 578 | 23.8 (4.5) | 23.4-24.2 | 0.028 |
| **Education**  Primary |  |  |  |  |  |  |  |  |  |  |
|  | 11 | 1.1 | 0.6-1.9 | 7 | 1.6 | 0.8-3.3 | 4 | 0.7 | 0.3-1.8 | 0.314 |
| Secondary (not matric) | 291 | 28.6 | 25.9-31.4 | 130 | 29.5 | 25.4-33.9 | 161 | 27.9 | 24.3-31.7 |  |
| Matric plus | 717 | 70.4 | 67.5-73.1 | 304 | 68.9 | 64.5-73.1 | 413 | 71.5 | 67.6-75.0 |  |
| **Current relationship status** |  |  |  |  |  |  |  |  |  |  |
| Currently not in a relationship | 183 | 18.0 | 15.7-20.5 | 97 | 22.0 | 18.4-26.1 | 86 | 14.9 | 12.2-18.1 | 0.014 |
| Currently married / Cohabiting | 69 | 6.8 | 5.4-8.5 | 29 | 6.6 | 4.6-9.3 | 40 | 6.9 | 5.1-9.3 |  |
| Currently dating (not cohabiting) | 766 | 75.3 | 72.5-77.8 | 315 | 71.4 | 67.0-75.5 | 451 | 78.2 | 74.6-81.4 |  |
| **Employed** |  |  |  |  |  |  |  |  |  |  |
| No | 832 | 81.7 | 79.1-83.9 | 332 | 75.3 | 71.0-79.1 | 500 | 86.5 | 83.5-89.1 | <0.001 |
| Yes | 187 | 18.4 | 16.1-20.9 | 109 | 24.7 | 20.9-29.0 | 78 | 13.5 | 10.9-16.5 |  |
| **Main source of income is a child support grant** |  |  |  |  |  |  |  |  |  |  |
| No | 733 | 71.9 | 69.1-74.6 | 342 | 77.6 | 73.4-81.2 | 391 | 67.7 | 63.7-71.3 | <0.001 |
| Yes | 286 | 28.1 | 25.4-30.9 | 99 | 22.5 | 18.8-26.6 | 187 | 32.4 | 28.7-36.3 |  |
| **Living area** |  |  |  |  |  |  |  |  |  |  |
| Urban-formal | 741 | 73.6 | 70.8-76.2 | 293 | 67.5 | 63.0-71.8 | 448 | 78.2 | 74.6-81.4 | <0.001 |
| Urban-informal | 166 | 16.5 | 14.3-18.9 | 89 | 20.5 | 17.0-24.6 | 77 | 13.4 | 10.9-16.5 |  |
| Rural | 100 | 9.9 | 8.2-11.9 | 52 | 12.0 | 9.2-15.4 | 48 | 8.4 | 6.4-10.9 |  |
| **Often or sometimes goes without food** |  |  |  |  |  |  |  |  |  |  |
| No | 849 | 83.4 | 81.0-85.6 | 366 | 83.2 | 79.4-86.4 | 483 | 83.6 | 80.3-86.4 | 0.871 |
| Yes | 169 | 16.6 | 14.4-19.0 | 74 | 16.8 | 13.6-20.6 | 95 | 16.4 | 13.6-19.7 |  |
| **Very difficult to get money in case of an emergency** |  |  |  |  |  |  |  |  |  |  |
| No | 674 | 66.1 | 63.2-69.0 | 301 | 68.3 | 63.8-72.4 | 373 | 64.5 | 60.5-68.3 | 0.214 |
| Yes | 345 | 33.9 | 31.0-36.8 | 140 | 31.8 | 27.6-36.2 | 205 | 35.5 | 31.7-39.5 |  |

ª Pearson’s chi square and t test were applied to compare the baseline socio-demographic characteristics between each exposure group

**Table S3: Baseline social demographic and behavioral characteristics of HIV negative women who were retained and lost to follow at 3-month visit with differential loss-to-follow-up by exposure group**

|  | Rape-exposed  (n=441) | | | Unexposed (Control Group)  (n = 578) | |  | Differential impact on loss to follow-up between groups |
| --- | --- | --- | --- | --- | --- | --- | --- |
|  | **Retained**  n=301 (68%) | **Lost to follow up**  n=140 (32%) | ***P* value ^a^** | **Retained**  n=484 (84%) | **Lost to follow up**  n=94 (16%) | ***P* value ^a^** | ***P* value ^c^** |
|  | n (%) or mean (SD) | |  | n (%) or mean (SD) | |  |  |
| Age (years) | 23∙3 (4∙8) | 22∙8 (4∙4) | 0∙220 | 24∙0 (4∙6) | 22∙9 (4∙2) | 0∙027 | 0∙358 |
| Education grade 12 and more | 206 (68∙4%) | 98 (70∙0%) | 0∙742 | 341 (70∙5%) | 72 (76∙6%) | 0∙228 | 0∙480 |
| Employed | 70 (23∙3%) | 39 (27∙9%) | 0∙297 | 65 (13∙4%) | 13 (13∙8%) | 0∙917 | 0∙604 |
| Currently in an intimate relationship* | 301 (77∙1%) | 112 (80∙0%) | 0∙490 | 407 (84∙3%) | 84 (89∙4%) | 0∙204 | 0∙527 |
| IPV | 186 (61∙8%) | 72 (51∙4%) | 0∙040 | 269 (55∙6%) | 40 (42∙6%) | 0∙021 | 0∙744 |
| NPSV* | 4∙5 (1∙0) | 4∙5 (1∙0) | 0∙682 | 4∙1 (0∙3) | 4∙1 (0∙3) | 0∙537 | 0∙626 |
| Relationship control scale | 20∙9 (6∙5) | 21∙3 (6∙5) | 0∙529 | 21∙5 (5∙3) | 22∙0 (5∙3) | 0∙360 | 0∙695 |
| HSV2 positive | 167 (55∙5%) | 72 (51∙4%) | 0∙426 | 264 (54∙6%) | 42 (44∙7%) | 0∙080 | 0∙446 |
| Transactional sex | 25 (8∙3%) | 14 (10∙0%) | 0∙560 | 42 (8∙7%) | 5 (5∙3%) | 0∙276 | 0∙224 |
| Multiple partners | 51 (16∙9%) | 25 (17∙9%) | 0∙813 | 53 (11%) | 9 (9∙6%) | 0∙693 | 0∙647 |
| STI ever* | 132 (43∙9%) | 46 (32∙9%) | 0∙028 | 190 (39∙3%) | 39 (41∙5%) | 0∙696 | 0∙076 |
| Social support*** | 35∙2 (5∙1) | 34∙8 (5∙9) | 0∙468 | 35∙0 (4∙6) | 36∙1 (4∙2) | 0∙041 | 0∙039 |
| Perceived stress** | 23∙0 (5∙9) | 23∙0 (6∙1) | 0∙981 | 21∙5 (5∙0) | 20∙3 (5∙0) | 0∙039 | 0∙095 |
| Previous experiences of trauma | 1∙9 (1∙7) | 1∙7 (1∙7) | 0∙112 | 1∙1 (1∙4) | 0∙8 (1∙2) | 0∙116 | 0∙693 |
| Childhood trauma | 16∙6 (3∙6) | 16∙6 (3∙9) | 0∙975 | 16∙0 (2∙8) | 15∙6 (2∙7) | 0∙204 | 0∙272 |
| Audit C score* | 2∙1 (2∙6) | 1∙7 (2∙3) | 0∙180 | 1∙6 (2∙3) | 1∙4 (2∙4) | 0∙727 | 0∙676 |
| PTSS-DTS* | 35∙3 (16∙0) | 33∙6 (15∙1) | 0∙292 | 6∙4 (9∙8) | 5∙3 (9∙2) | 0∙284 | 0∙634 |
| Depression-CESD* | 33∙2 (12∙7) | 33∙2 (12∙3) | 0∙973 | 13∙3 (9∙1) | 11∙8 (8∙6) | 0∙157 | 0∙233 |
| Received PEP ¶ | 196 (94∙2) | 32 (94∙1) | 0∙979 |  | | | |
| Adherence to PEP ¶¶ | 177 (90∙3) | 30 (93∙8) | 0∙532 |  | | | |

IPV= intimate partner violence-ever experienced emotional, economical, sexual or physical IPV. HSV2=herpes simplex virus type 2. NPSV=non-partner sexual violence.

Transactional sex=with a casual partner. Multiple partners= 2 or more partners in the last year. STI Ever= Been told by a health worker that they had an STI and have had vaginal discharge or an ulcer. PTSS-Post Traumatic Stress Syndrome, DTS- Davidson trauma scale. CESD-Center for Epidemiological Studies Depression. PEP-post-exposure prophylaxis

*N=1018, with retained total n=784 and unexposed retained n=483. **N=1017, with retained total n=783 and unexposed retained n=482. ***N=1016, with retained total n=782 and unexposed retained n=481 ¶N=242, with retained total n=208 and lost to follow up total n=34 ¶¶ Among the participants that received PEP, with retained n=196 and lost to follow n=32

Loss to follow-up associated with exposure group (*p*-value=< 0∙000)

ª Pearson’s chi-square and t-test were applied to compare the baseline characteristics between the retained and the loss to follow up within each exposure group

ͨ The logistic regression was applied to obtain the interaction term effect of the baseline characteristic and the exposure group on the loss to follow up outcome

**Table S4: Baseline social demographic and behavioural characteristics of HIV negative women who were retained and lost to follow at 9-month visit with differential loss-to-follow-up by exposure group**

|  | Rape-exposed (n=441) | | | Unexposed (Control Group) (n=578) | | | Differential impact on loss to  follow-up between groups |
| --- | --- | --- | --- | --- | --- | --- | --- |
|  | **Retained**  n=262 (59%) | **Lost to follow up**  n=179 (41%) | ***P* Value^a^** | **Retained**  n=452 (78%) | **Lost to follow up**  n=126 (22%) | ***P* Value ^a^** | ***P* Value ^c^** |
|  | n (%) or mean (SD) | |  | n (%) or mean (SD) | |  |  |
| Age (years) | 23.1 (4.7) | 23.2 (4.5) | 0.819 | 24.0 (4.6) | 23.2 (4.1) | 0.072 | 0.133 |
| Education grade 12 and more | 178 (67.9%) | 126 (70.4%) | 0.585 | 320 (70.8%) | 93 (73.8%) | 0.508 | 0.909 |
| Employed | 60 (22.9%) | 49 (27.4%) | 0.285 | 61 (13.5%) | 17 (13.5%) | 0.999 | 0.519 |
| Currently in an intimate relationship* | 207 (79.0%) | 137 (76.5%) | 0.538 | 380 (84.3%) | 111 (88.1%) | 0.285 | 0.222 |
| IPV | 162 (61.8%) | 96 (53.6%) | 0.086 | 251 (55.5%) | 58 (46.0%) | 0.059 | 0.875 |
| NPSV* | 4.5 (1.0) | 4.5 (1.0) | 0.932 | 4.1 (0.3) | 4.1 (0.3) | 0.515 | 0.549 |
| Relationship control scale | 21.0 (6.2) | 21.0 (7.0) | 0.912 | 21.4 (5.3) | 22.0 (5.3) | 0.275 | 0.343 |
| HSV2 positive | 146 (55.7%) | 93 (52.0%) | 0.435 | 248 (54.9%) | 58 (46.0%) | 0.079 | 0.470 |
| Transactional sex | 22 (8.4%) | 17 (9.5%) | 0.689 | 40 (8.9%) | 7 (5.6%) | 0.232 | 0.240 |
| Multiple partners | 46 (17.6%) | 30 (16.8%) | 0.828 | 50 (11.1%) | 12 (9.5%) | 0.622 | 0.795 |
| STI ever* | 113 (43.1%) | 65 (36.3%) | 0.152 | 175 (38.8%) | 54 (42.9%) | 0.411 | 0.112 |
| Social support*** | 35.3 (5.0) | 34.7 (5.9) | 0.261 | 34.9 (4.6) | 36.1 (4.4) | 0.013 | 0.009 |
| Perceived stress** | 22.7 (5.6) | 23.5 (6.4) | 0.207 | 21.6 (5.1) | 20.5 (4.9) | 0.031 | 0.013 |
| Previous experiences of trauma | 1.9 (1.6) | 1.8 (1.7) | 0.654 | 1.1 (1.4) | 0.9 (1.2) | 0.408 | 0.696 |
| Childhood trauma | 16.5 (3.5) | 16.8 (4.0) | 0.488 | 16.0 (2.8) | 15.7 (2.8) | 0.379 | 0.264 |
| Audit C score* | 2.1 (2.6) | 1.9 (2.4) | 0.531 | 1.6 (2.3) | 1.4 (2.2) | 0.335 | 0.743 |
| PTSS-DTS* | 35.1 (15.8) | 34.1 (15.7) | 0.518 | 6.5 (9.8) | 5.5 (9.1) | 0.314 | 0.571 |
| Depression-CESD* | 33.2 (12.6) | 33.2 (12.6) | 0.997 | 13.3 (9.1) | 12.1 (8.5) | 0.189 | 0.271 |

IPV= intimate partner violence-ever experienced emotional, economical, sexual or physical IPV. HSV2=herpes simplex virus type 2. NPSV=non-partner sexual violence.

Transactional sex= sex with a casual partner. Multiple partners=2 or more partners in the last year. STI ever= Been told by a health worker that they had an STI and have had vaginal discharge or ulcer. PTSS-Post Traumatic Stress Syndrome. DTS- Davidson trauma scale. CESD-Center for Epidemiological Studies Depression

*N=1018, with retained total n=713 and unexposed retained n=451. **N=1017, with retained total n=712 and unexposed retained n=450. ***N=1016, with retained total n=711 and unexposed retained n=449.

Loss to follow-up is associated with exposure group (*p* value= <0∙000)

ª Pearson’s chi square and t test were applied to compare the baseline characteristics between the retained and the loss to follow up within each exposure group

ͨ The logistic regression was applied to obtain the interaction term effect of the baseline characteristic and the exposure group on the loss to follow up outcome

**Table S5: Weibull model of relative incidence of HIV among rape exposed women compared to non-rape exposed women a****djusted for variables associated with drop-out, baseline covariates and time varying covariates (N=845).**

| Unadjusted model (N=845) | | |
| --- | --- | --- |
|  | **Hazard Ratio (95%CI)** | ***P* value** |
| Unexposed (Control Group) | 1.00 | .. |
| Rape Exposed | 1∙46 (0∙95-2∙24) | 0∙083 |
| Models adjusted for baseline covariates (N=845) | | |
| *Adjusted for age* | | |
| Unexposed (Control Group) | 1∙00 | .. |
| Rape Exposed | 1∙43 (0∙93-2∙20) | 0∙102 |
| *Adjusted for age and previous trauma experience* | |  |
| Unexposed (Control Group) | 1∙00 | .. |
| Rape Exposed | 1∙51 (0∙97-2∙35) | 0∙070 |
| Models adjusted for baseline (age and previous trauma experiences) and time-varying covariates (N=845) | | |
| *Adjusted for baseline and time varying covariates: multiple partners in follow-up period** | | |
| Unexposed (Control Group) | 1∙00 | .. |
| Rape Exposed | 1∙58 (1∙01-2∙46) | 0∙044 |
| *Adjusted for baseline and time varying covariates: transactional sex with casual partner in follow-up period *** | | |
| Unexposed (Control Group) | 1∙00 | .. |
| Rape Exposed | 1∙62 (1∙04 -2∙52) | 0∙034 |
| *Adjusted for baseline and time varying covariates: combined multiple partners and transactional sex with casual partner (3 levels) in follow-up period **** | | |
| Unexposed (Control Group) | 1∙00 | .. |
| Rape Exposed | 1∙61 (1∙03-2∙50) | 0∙037 |
| Model adjusted for baseline variables (age and previous trauma experiences) and variables associated with drop out (social support and perceived stress) and time varying covariate (combined multiple partners in past year and transactional sex with casual partner) (N=842) | | |
| Unexposed (Control Group) | 1∙00 | .. |
| Rape Exposed | 1∙61 (1∙03-2∙51) | 0∙037 |

*Reported having two or more sexual partners in the period between interviews

** Reported having transactional sex with a casual partner in the period between interviews

*** A three levels composite sexual risk behavior variable (low, moderate and high) based on combining multiple partners and transactional sex with casual partner in the follow-up period

**Table S6:** **Cox regression and Weibull model for HIV incidence following rape exposure adjusted for variables associated with drop-out, baseline covariates and time varying covariates: showing models with all participants and models excluding control participants with previous rape exposure**

|  | Models among all HIV negative participants | | | | | Models excluding HIV negative participants with previous rape exposure* | | | | |
| --- | --- | --- | --- | --- | --- | --- | --- | --- | --- | --- |
|  | **COX Regression** | | | **Weibull** | | **COX Regression** | | | **Weibull** | |
|  | n | Hazard Ratio (95%CI) | *P* value | Hazard Ratio (95%CI) | *P* value | n | Hazard Ratio (95%CI) | *P* value | Hazard Ratio (95%CI) | *P* value |
| All participants retained after baseline | 842 | 1∙59 (1∙01-2∙48) | 0∙043 | 1∙61 (1∙03-2∙51) | 0∙037 | 800 | 1∙50 (0∙95-2∙36) | 0∙080 | 1∙52 (0∙96-2∙38) | 0∙071 |
| Retained from Month 3 | 781 | 1∙61 (1∙03-2∙51) | 0∙038 | 1∙62 (1∙04-2∙53) | 0∙035 | 740 | 1∙52 (0∙96-2∙39) | 0∙072 | 1∙53 (0∙97-2∙40) | 0∙067 |
| Retained from Month 6 | 740 | 1∙62 (1∙03-2∙53) | 0∙036 | 1∙63 (1∙04-2∙54) | 0∙033 | 701 | 1∙53 (0∙97-2∙41) | 0∙067 | 1∙54 (0∙98-2∙41) | 0∙063 |
| Retained from Month 9 | 714 | 1∙63 (1∙04-2∙55) | 0∙032 | 1∙64 (1∙05-2∙56) | 0∙031 | 677 | 1∙54 (0∙98-2∙43) | 0∙060 | 1∙55 (0∙98-2∙43) | 0∙059 |

*exclude 94 participants in control group who reported previous rape exposure

**Table S7: Cox regression model of relative incidence of HIV among rape exposed women compared to non-exposed start time shifted to month 3 (N=773):** **adjusted for variables associated with drop-out, baseline time varying covariates**

|  | Hazard Ratio (95%CI) | *P* value |
| --- | --- | --- |
| Unadjusted model (N=773) | | |
| Unexposed | 1.00 | .. |
| Rape Exposed | 1.48 (0.94 - 2.34) | 0.089 |
| Adjusted for baseline covariates (N=773) | | |
| *Adjusted for baseline age* | | |
| Unexposed | 1.00 | .. |
| Rape Exposed | 1.47 (0.93 - 2.32) | 0.099 |
| *Adjusted for baseline age and previous trauma experience reported at baseline* | | |
| Unexposed | 1.00 | .. |
| Rape Exposed | 1.60 (1.00 - 2.57) | 0.051 |
| Adjusted for baseline covariates (age and previous trauma experiences) and time-varying covariates (N=773) | | |
| *Adjusting for baseline covariates and time varying covariate: multiple partners in follow-up period ** | | |
| Unexposed | 1.00 | .. |
| Rape Exposed | 1.68 (1.05 - 2.69) | 0.032 |
| *Adjusting for baseline covariates and time varying covariate: transactional sex with casual partner in follow-up period *** | | |
| Unexposed | 1.00 | .. |
| Rape Exposed | 1.72 (1.07 - 2.76) | 0.024 |
| *Adjusting for baseline and time varying covariates: combined measure of multiple partners and transactional sex with casual partner (3 levels) **** | | |
| Unexposed | 1.00 | .. |
| Rape Exposed | 1.70 (1.06 - 2.72) | 0.028 |
| Adjusted for baseline covariates (age and previous trauma experiences) and variables associated with drop out (social support and perceived stress) and time varying covariates (combined multiple partners in past year and transactional sex with casual partner) (N=770) | | |
| Unexposed | 1.00 | .. |
| Rape Exposed | 1.71 (1.06 - 2.74) | 0.028 |

*Reported having two or more sexual partners in the period between interviews

** Reported having transactional sex with a casual partner in the period between interviews

*** A three levels composite sexual risk behavior variable (low, moderate and high) based on combining multiple partners and transactional sex with casual partner in the follow-up period
