# Supplementary material for: Increase in HIV incidence in women exposed to rape
Source: AIDS. 2020 Dec 1;35(4):633–42. doi: 10.1097/QAD.0000000000002779 (PMC7924974; doi:10.1097/QAD.0000000000002779)
Supplement: Supplemental Digital Content [file aids-35-633-s004.docx]

**Table S1. Incidence rates for person-years pre-12 months and post-12 months follow-up by exposure group**

|  | **Rape-exposed** | | | **Unexposed (Control Group)** | | |
| --- | --- | --- | --- | --- | --- | --- |
| **Time** | **Person-years** | **HIV infection** | **Incidence rate per 100 person-years (95% CI)** | **Person-years** | **HIV infection** | **Incidence rate per 100 person-years (95% CI)** |
| Month 3 to Month 12 | 286∙75 | 20 | 7∙0 (4∙5 - 10∙8) | 469∙75 | 22 | 4∙7 (3∙1 - 7∙1) |
| Month 12 to Month 36 | 272∙50 | 17 | 6∙2 (3∙9 - 10∙0) | 576∙50 | 27 | 4∙7 (3∙2 - 6∙8) |
| Total | 559∙25 | 37 | 6∙6 (4∙8 - 9∙1) | 1046∙25 | 49 | 4∙7 (3∙5 - 6∙2) |
